# How to use biogas?: A systematic review of biogas utilization pathways and business models

**DOI:** 10.1186/s40643-022-00545-z

**Published:** 2022-05-28

**Authors:** Anica Mertins, Tim Wawer

**Affiliations:** 1 Osnabrück University of Applied Sciences, Osnabrück, Germany; 2 Osnabrück University of Applied Sciences, Lingen (Ems), Germany

**Keywords:** Biogas, Business models, Utilization pathways, CHP, Direct usage, Biogas upgrading, Hydrogen from biomass

## Abstract

There are many options for the utilization of biogas in different energy sectors (power, heat, mobility). The technical possibilities of using biogas are more diverse than the actual business models applied in the biogas industry. This paper shows the possible utilization pathways of biogas, divided into coupled power and heat generation, direct utilization and upgrading to a gas of a higher value. Subsequently, an overview of the business models discussed is given by a systematic literature review. The latter shows that the investigation of biogas business models is focused mainly on the last decade and has increased slightly over time. The regions of investigation can be found worldwide, with a clear focus on Europe. Direct use is studied mainly in the Asian and African regions. In the European context, a shift from investigating combined heat and power use to upgrading the biogas produced is evident.

## Introduction 

Over 90% of the biogas produced in the world was used for the production of power and heat in 2018, with only the remaining 9% being used as biomethane in the mobility sector or for injection into the natural gas grid (International Energy Agency 2020). However, the increasing number of biomethane plants suggests that the share of usage as biomethane will increase in the future (Banja et al. 2019). The utilization pathways differ between the countries, depending very much on the framework conditions of the country in which the biogas plant is operated (Capodaglio et al. 2016). Biogas plants in different countries have developed differently in terms of the substrate usage, pretreatment technology, plant size and utilization pathway of the biogas (Stürmer et al. 2021a). Several regions throughout the world are striving for climate neutrality based on an energy system supplied 100% from renewable energies. This means that not only the power supply, but also the heat and fuel supply have to abandon fossil fuels. This raises the question what the future will look like and what role biogas technology will play in it.

In this paper, the utilization pathway is defined as a technical possibility for the use of biogas. This includes particularly converting biogas into usable energy and the subsequent possible fields of application. The term ‘business model’ is used to describe the options for monetizing biogas. In addition to the revenue streams, the business model specifies the utilization pathway and the profitability in a sales market, including costs.

This paper presents a systematic review of utilization pathways for biogas and evaluates the business models associated with each pathway. Conclusions can be drawn about the temporal course of research interest and geographic characteristics. The various business models that are conceivable based on the utilization pathways are presented. This approach reveals differences between the technical feasibility of a usage pathway and its actual implementation as part of a business model. These business models are examined independently of any specific region and should, therefore, be considered independently of local funding programs.

Several publications have already examined and compared different pathways of biogas, for example (Bystricky et al. 2010; Patrizio et al. 2015; Wu et al. 2016). Exemplarily, the use of biogas in a decentralized combined heat and power (CHP) plant, the injection of upgraded biomethane into the gas grid for off-site power and heat generation, and the use of biomethane as fuel are all under investigation. Business models for biogas have also already been investigated in the literature (Heffels et al. 2012; Horschig et al. 2019; Karlsson 2019). Focuses are, for example, on the direct marketing of power or different possibilities for biomethane becoming advantageous as a business model. To the best of our knowledge, no comprehensive analysis of biogas business models in the different pathways exists in the current literature. The novelty of this paper is, therefore, the analysis of the technical possibilities for the use of biogas combined with a detailed presentation of possible business models that are discussed in the literature. The specifics of each business model are discussed and the research strength in the area of the different business models is shown. In the context of the transformation of the energy system, it is relevant to reveal technical possibilities for energy generation that enable a profitability. The connection of technical utilization pathways with the consideration of possible business models plays a central role in the design of future energy supply, such as the use of biogas.

The paper is structured as follows: firstly, an overview of existing technical possibilities for biogas utilization is given. Subsequently, the method of literature review is presented. The results of the literature research on biogas business models are then presented in detail, subdivided according to the different utilization routes. The discussion of the results is followed by a conclusion.

### Biogas utilization pathways

Biogas is a renewable energy that can be used in various ways, which is a major advantage of the technology. Possible utilization pathways are conceivable in all areas of consumption (Capodaglio et al. 2016), with three main distinctions:

1. Using the biogas in a CHP unit;
2. Using the biogas directly, for example, in machines or facilities in agricultural operations; and
3. Upgrading the biogas to a gas of a higher value.

**Fig. 1:**
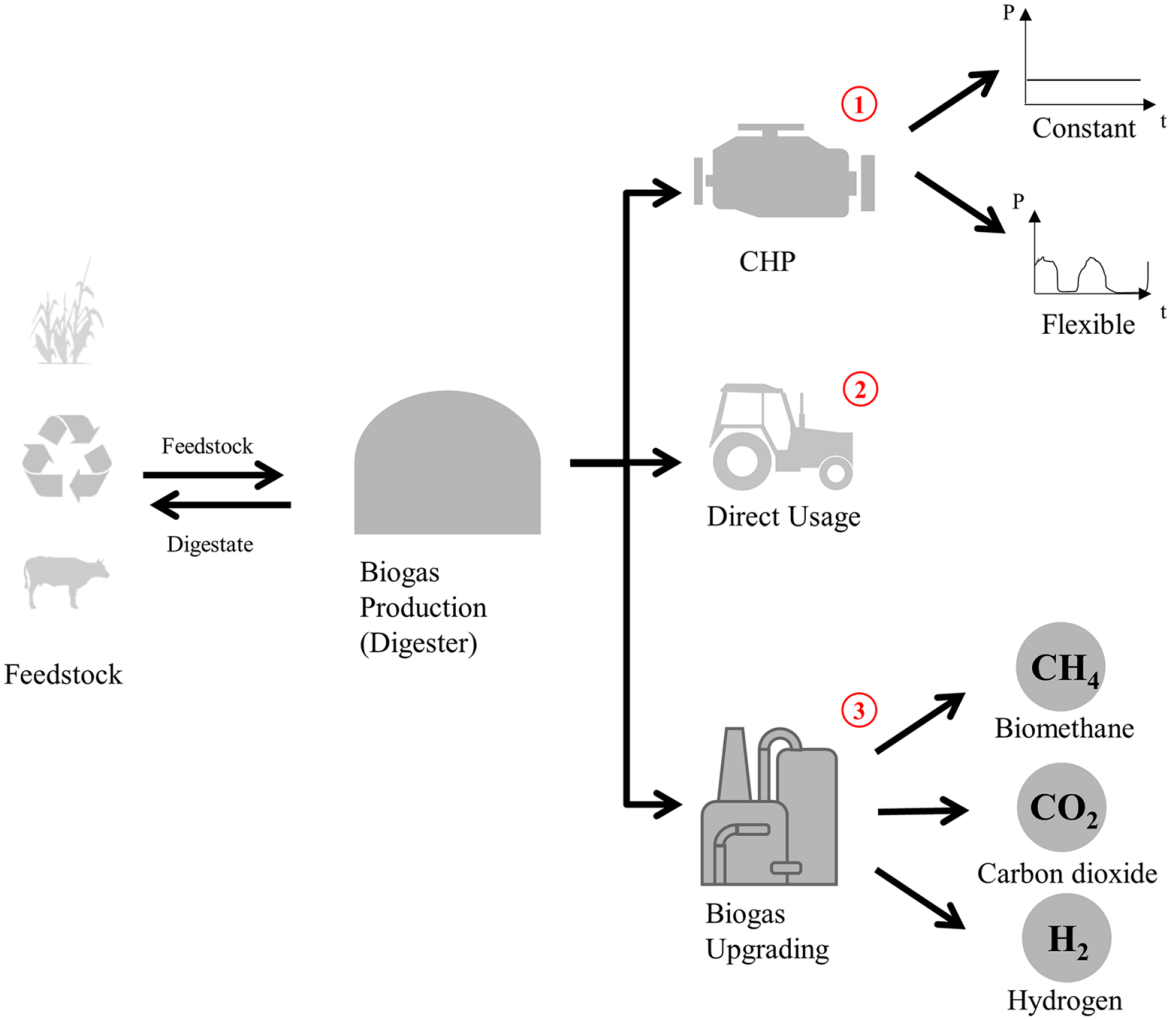
Potential utilization pathways for biogas

The different pathways are shown in Fig. 1 and are explained in detail below.

### Pathway 1: CHP generation

The most commonly used pathway for biogas is in decentralized CHP units. The biogas produced is first dried, desulfurized and then used in a gas engine that drives a generator delivering power and heat. The power is normally used externally and, therefore, fed into the grid. Part of the generated heat is needed to operate the digester. The remaining heat can be used externally, for example, to heat buildings or for crop drying (Watter 2019). The electrical efficiency is usually between 30 and 40%, and the corresponding thermal efficiency is 35 to 55% (Lantz 2012). The highest mean fuel efficiency is achieved when all the heat produced can be used all year round (Fischer et al. 2016; Watter 2019).

A CHP unit can be operated in a base load mode, i.e., generates a constant amount of power and heat, or in a flexible mode. The latter enables the energy generation to be adapted to a demand for power or heat, or is driven by price signals. The digester is designed for a constant output, therefore, the flexibilization requires the adaptation of components (Liebetrau et al. 2015).

Regarding existing biogas plants, which were originally designed for base load operation, two retrofitting options for flexible generation are available: they can be adapted for flexible power production either by lowering the rated output or by increasing the power generation capacity. For the latter, a second CHP unit, a larger gas storage tank, facilities for external heat utilization and an adaptation of the connection to the power grid (e.g., transformer) are required. Alternative to the expansion of the gas storage, flexible biogas production can be achieved by changing the feedstock-feeding management (Daniel-Gromke et al. 2019).

One advantage of on-site use of a CHP unit is that the energy generated can be used to cover the internal energy demand of the anaerobic digestion process. The power demand for the digester and ancillary components in other utilization pathways must be covered from the power grid. Heat, on the other hand, can be generated by a gas boiler (Goulding and Power 2013).

### Pathway 2: direct usage of biogas

In the second pathway, biogas is used in the immediate vicinity of the biogas plant. The aim of the on-site use of biogas is to reduce energy procurement costs or to reach energy self-sufficiency. Biogas can be used directly in households (Hamid and Blanchard 2018), for uncoupled heat generation, e.g., in heat boilers for use as process heat or steam or as fuel for machines on the farm (Lampinen 2004).

Another option is the direct utilization of biogas in tractors or agricultural machinery. Currently, biogas is not used in common practice for mobile internal combustion engines without upgrading (Owczuk et al. 2019). As untreated biogas does not meet the minimum specifications of a compressed natural gas (CNG) fuel due to the comparatively high proportion of carbon dioxide (CO_2_), water vapor and hydrogen sulfide, it cannot be used in CNG-powered vehicles (Kruczyński et al. 2013). Compared to biomethane or natural gas, the energy content of biogas per unit mass is significantly lower. Large quantities of biogas can, thus, only be stored under high pressure, with the upgrading, storage and handling in this case leading to increased costs (Mihic 2004). On the other hand, biogas that has been separated from hydrogen sulfide and water is a significantly cheaper fuel than biomethane (Kruczyński et al. 2013). Another difficulty of using pure biogas as a fuel is to ensure a secure supply. Thus, periods with low biogas production or high consumption must be balanced out by storage (Redwanz and Walter 1984).

However, the use of biogas in a dual-fuel engine, together with the use of diesel, has been investigated. Different parameters are focused on here, for example, the concentration of different exhaust gases (Jaber et al. 2009; Owczuk et al. 2019), the effect of different fuel mixing ratios (Matuszewska et al. 2016). (Lampinen 2004) reported on a farm that is completely self-sufficient in energy (power, heat and fuel, and also fertilizers) through the biogas plant. Tractors using biogas as fuel were introduced in the early 2010s. The dual use of biogas and biodiesel to compensate for the low energy density of biogas saves up to 40% in fuel costs, according to one manufacturer (Kruczyński et al. 2013).

### Pathway 3: upgrading of biogas to a gas of higher value

The third pathway is upgrading biogas to a gas of a higher value. Upgrading of biogas to biomethane is considered a mature technology (Verotti et al. 2016). The upgrading process mainly involves the separation of various impurities from the biogas. The most important part of the upgrading process is the separation of CO_2_, with the aim of increasing the methane content of the biogas (Bragança et al. 2020). The order of the individual steps for upgrading depends on the gas properties and the method of capturing the CO_2_ (Deutsche Energie-Agentur GmbH 2019a). Upgrading methods are water scrubbing, chemical scrubbing, physical scrubbing, pressure swing adsorption and membrane separation (Miltner et al. 2017). Biomethane provides the advantage of multiple applicability, thus, it can be used as a fuel in the mobility sector, as a natural gas substitute in the heating sector, or off-site in a CHP unit. Upgrading leads to higher costs, higher energy consumption and more consumption of material. In addition, methane slip can worsen the environmental footprint (Schmid et al. 2019).

The upgrading allows the injection of the gas into the existing gas grid. This provides the advantage that it can be used spatially and temporally independently of its production. This can help to make the use more efficient and, thus, increase sustainability, as a result of demand-orientated energy generation (Budzianowski and Budzianowska 2015). Another advantage is that the infrastructure for distributing and using the biomethane has already been established (Cavana and Leone 2019). Feeding biomethane into the grid is already a common practice in countries such as Germany, Sweden, the Netherlands, Switzerland and Austria (Korberg et al. 2020). The upgrading of biogas to biomethane becomes cheaper with increasing biogas volumes due to cost degression in investments costs; therefore, this pathway is particularly interesting for large biogas plants. There is the possibility of plant pooling, so that smaller biogas plants can also take advantage of the cost degression. Other biogas plants in the vicinity, which can cooperate for joint upgrading, are essential for this (Dotzauer et al. 2021). Proximity to the natural gas grid is important for the success of this utilization pathway (Pasini et al. 2019). One way to implement pooling is to sell raw biogas to the operator of the upgrading plant. This requires the construction of microgrids to connect the biogas plants to the upgrading plant. In this scenario, the biogas plant operator is therefore only responsible for the production of the biogas, which is subsequently upgraded by another market participant (Dotzauer et al. 2021).

In addition to upgrading following the biogas production (ex situ), it is also possible to produce biomethane in the digester, so-called in situ processes. Examples are CO_2_ desorption, the pressure reactor, H_2_ (hydrogen) addition and electromethanogenesis. For H_2_ addition, for example, the H_2_ is fed into the digester. The aim is to achieve biomethane of natural gas quality directly with the help of bacteria inside the digester (Aryal et al. 2018). Initial studies indicate that in situ methods can offer better profitability for small to medium-sized plants and achieve a methane content of 85%. However, in situ methods are still underdeveloped and currently mostly take place on a laboratory or microscale. Above all, research into the technology on a large scale needs to be improved (Sarker et al. 2018).

There is a possibility to further use the captured CO_2_, which is currently not a common practice (Billig et al. 2019). The biogenic CO_2_ does not cause any climate-relevant emissions and is suitable for various utilization pathways itself (van Basshuysen 2016). Possible areas of usage would be the food, chemical and pharmaceutical industries (Horschig et al. 2019) or the production of synthetic fuels (Eggemann et al. 2020). Capture and subsequent storage of CO_2_, known as Bioenergy with Carbon Capture & Storage (BECCS), is one option for making biogas production CO_2_-negative (Rosa et al. 2021).

Under certain circumstances, it may be necessary to adjust the calorific value of the biomethane (e.g., by adding liquid gas). The biomethane must be compressed according to the pressure level of the grid for injection into the natural gas grid. In summary, regarding injection, the quality of the gas must comply with the provisions of the gas class and the deviations must not exceed the permissible limits (Deutsche Energie-Agentur GmbH 2019a).

Another technical option is to upgrade biogas to green H_2_. Technical options for realization include reforming (steam, partial oxidation and autothermal reforming), biological processes (bio-photolysis, dark fermentation and photofermentation), thermochemical processes (pyrolysis, gasification, combustion and liquefaction) or water splitting (electrolysis, thermolysis and photolysis) (Nikolaidis and Poullikkas 2017). The advantage of reformation is that it is a proven technique, as it is conventionally an established process to produce syngas from natural gas (Wünning 2021).

## Material and methods 

A systematic literature analysis of possible business models and economically considered utilization pathways was carried out to obtain an overview of the established business models in the biogas industry. The EBSCOhost database was used for the systematic literature review. The main search term “biogas” had to be included in the title of all candidate papers. Various additional search keywords were used that had to be included in the title or abstract of the paper. The search terms include “business model”/“business case”/“economic”, “use”/“utilization”/“usage” and “pathway”/“path” in various combinations. The search was conducted between September 22 and October 18, 2021.

As a result of the systematic literature review, 242 scientific publications were found; after sorting out duplicate papers, 192 publications remained. The aim of the paper was determined by reviewing the title, abstract and keywords. On this basis, the relevance of the paper was determined for further investigation. Relevant for the selection of a publication was the clear consideration of the profitability of a business model. Publications addressing technical utilization paths or the ecological assessment were not considered further. Regarding the relevant papers, the literature utilized (backward search) and further developments in the research field (forward search) were examined for additional relevant publications. In conclusion, a set of 72 relevant papers remained for the detailed analysis.

**Fig.2:**
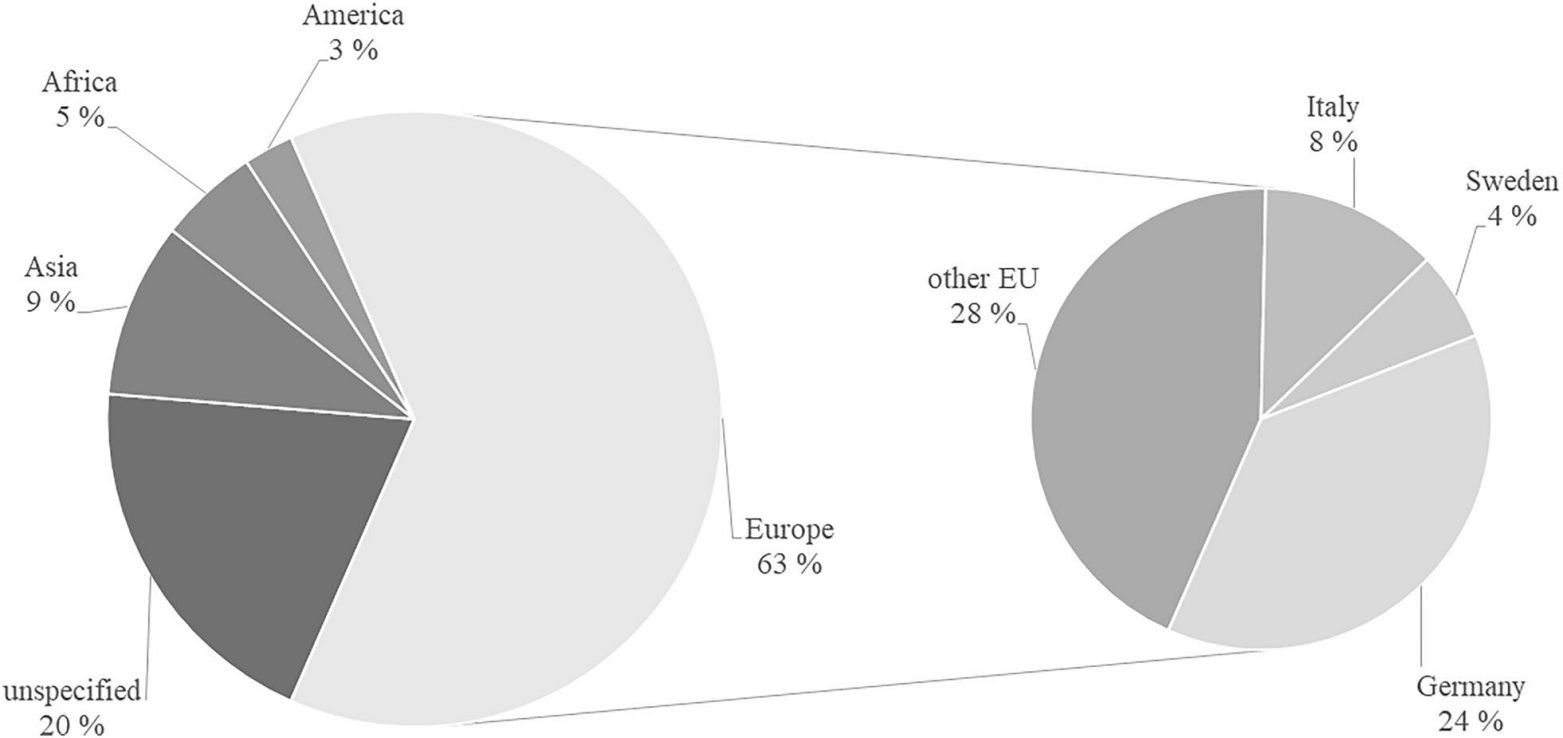
Distribution of papers by countries and regions considered

Figure 2 shows that interest in biogas business models and utilization pathways is mainly centered in the European region (64% of the relevant papers). Most articles in Europe deal with Italy, Sweden and Germany. A good third of the papers do not deal with any specific country in their methodology.

An initial classification of the publications into the three main biogas pathways: CHP usage, direct use or upgrading to a higher quality gas was made. If papers deal with several of these topics, they were included in more than one category. The papers in the three categories were examined regarding their publication date and the region under consideration and then classified again. The aim here is an evaluation according to the chronological course and geographical characteristics of individual usage pathways.

Different utilization pathways are covered in the papers, with a focus on CHP usage (*n* = 30). Another utilization pathway that has been studied frequently is biomethane (*n* = 26), with a focus on fuel production (*n* = 16) and CHP off-site usage (*n* = 6). In this context, the use of captured CO_2_ was also investigated in seven papers. Five papers investigated the use of biogas to produce H_2_.

**Fig. 3:**
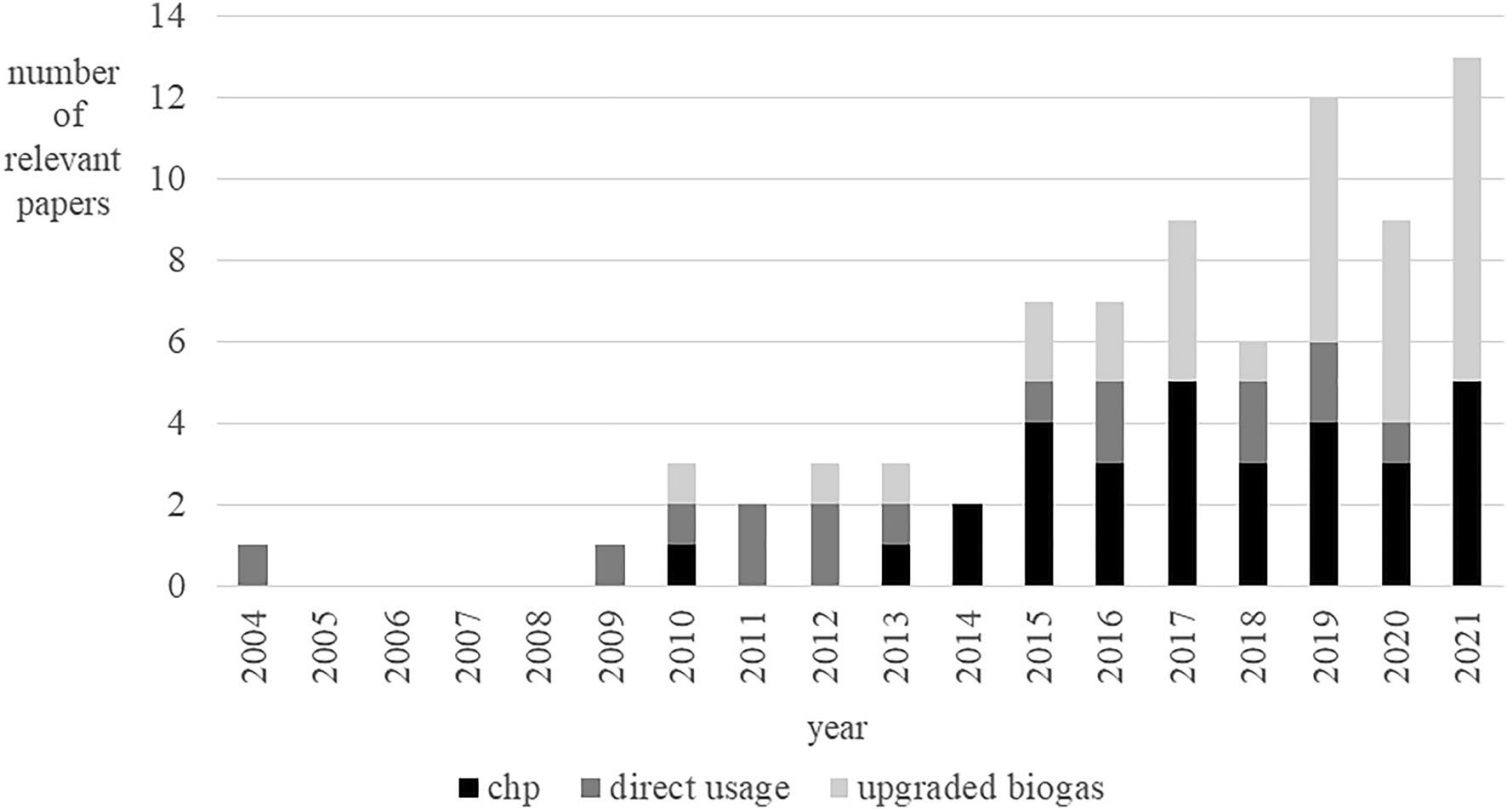
Development of relevant published papers by utilization pathway over time

Figure 3 shows that the number of relevant papers has increased over the last few years. The research interest of direct utilization of biogas has remained relatively constant over time. The interest in CHP use has increased significantly until 2015, which has shifted over time to the field of biogas upgrading. While almost 40% of the publications address the CHP business model, as well as the upgrading of biogas, the share of direct use is comparatively low at 20%.

The CHP generation is currently the biogas application pathway most widely discussed. It is treated in many publications in the literature. Thirty of the relevant 72 scientific papers deal with this utilization pathway. The first publication on biogas CHP usages dates back to 2010 (Bystricky et al. 2010), and since 2013, there has been an increasing number of publications in this field (Hahn et al. 2014; Szarka et al. 2013). The number of publications has been relatively constant since 2015. Research interest is concentrated in Europe, especially Germany (*n* = 15, e.g., (Butemann and Schimmelpfeng 2020; Theuerl et al. 2019)), Italy (*n* = 3, including (Gandiglio et al. 2016; Patrizio et al. 2017)) and Austria (*n* = 3, including (Saracevic et al. 2018; Stürmer et al. 2021b)).

**Fig. 4:**
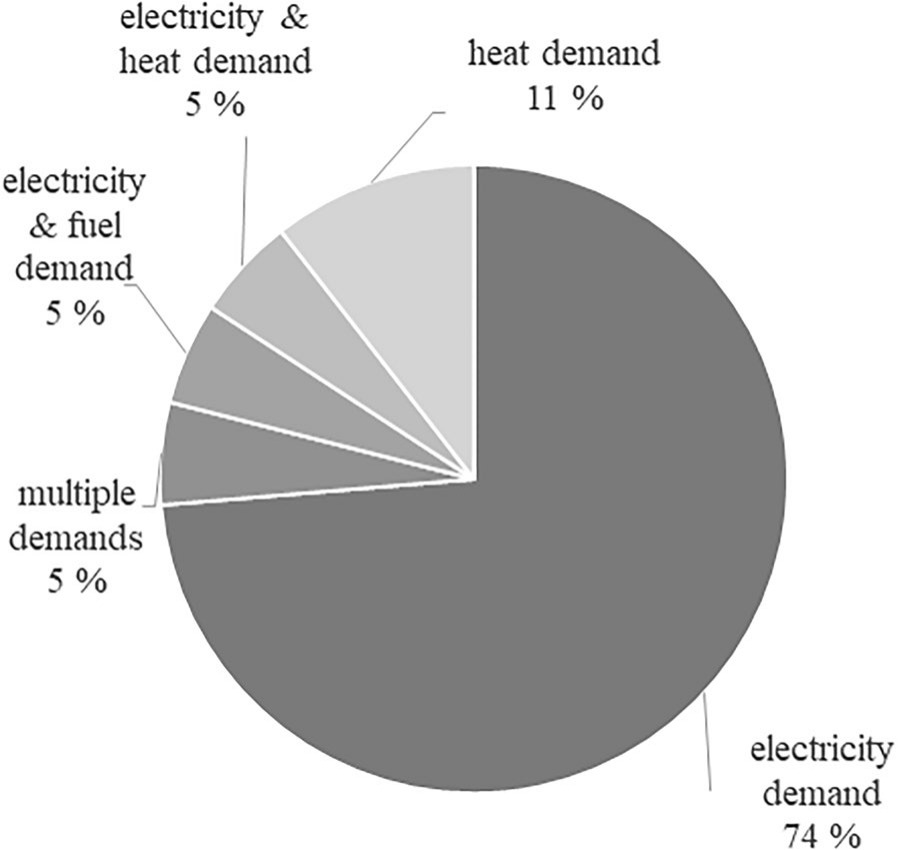
Aim of flexibilization of CHP plants investigated in studies, *n* = 19

**Fig. 5:**
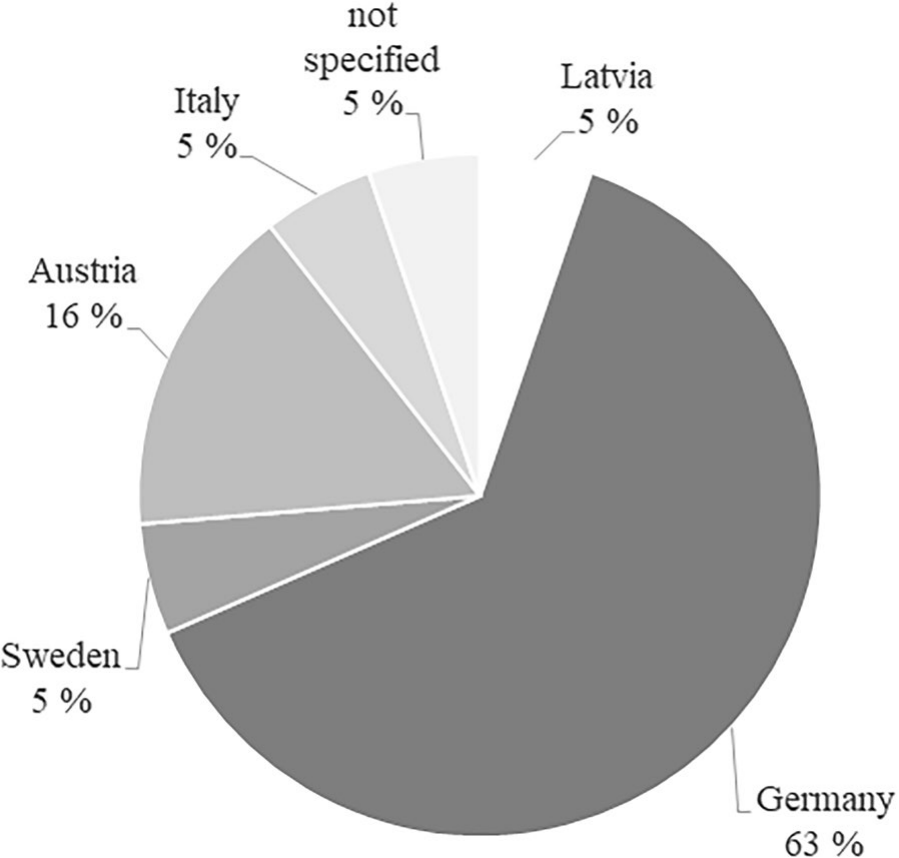
Allocation by country for studies on CHP flexibilization, *n* = 19

The aim of flexibilization of CHP units is primarily the power demand-orientated mode of operation, although various utilization pathways or mixed forms are also considered (Fig. 4). Hahn et al. (2014) and Häring et al. (2017) investigated the various options for flexible power production from biogas with the aim of meeting the requirements and demands of the power market. Grim et al. (2015) also compared the on-demand production of power to baseload operation and evaluates technical requirements and economic impacts of on-demand production. Dzene and Romagnoli (2015) showed the possibilities of using biogas to balance the volatile feed-in from wind energy, whereas Bär et al. (2020) pursued the goal of creating synergy effects from photovoltaics and biogas plants, with the aim of achieving a second-by-second balancing of volatile generation. The first publications on the subject of flexibilization deal with flexible production in Germany (Hahn et al. 2014, 2015; Szarka et al. 2013), as incentives have been set to produce power demand-orientated in the EEG (Renewable Energy Act) 2012. However, the research interest in flexibilization in other countries started in 2015, for example, in Latvia (Dzene et al. 2014) and Sweden (Grim et al. 2015). Research interest in this utilization pathway exists primarily in Germany, as more than half of the publications present the utilization pathway using the German example (Fig. 5) (Güsewell et al. 2021; Häring et al. 2017; Hijazi et al. 2019).

The direct use of biogas has been studied regularly in the literature since 2004. Seventeen relevant scientific publications were found in the literature search. They can be divided into two categories: the use of biogas in households, for example, for cooking or heating (*n* = 10), or in tractors or agricultural machinery (*n* = 7). The biogas for use in households is normally produced in a decentralized manner and used directly, for example, for cooking (Hamid and Blanchard 2018), but also to supply power or produce fertilizer for agricultural purposes (Das et al. 2016). Another goal of decentralized plants can be waste disposal, for example, of wastewater (Bensah and Brew-Hammond 2010) or to reduce greenhouse gas (GHG) emissions (Yuan et al. 2015). Research interest in biogas plants for household use is concentrated in Africa (Kenya (Hamid and Blanchard 2018), Ghana (Bensah et al. 2011; Bensah and Brew-Hammond 2010) and Africa in general (Kemausuor et al. 2018)), and Asia (Bangladesh (Das et al. 2016), China (Chen et al. 2012; Yuan et al. 2015), Indonesia (Silaen et al. 2020), Bali (Bößner et al. 2019) and Pakistan (Yasmin and Grundmann 2019)).

Twenty-six relevant papers were identified for the biogas upgrading pathway. The first publications date back to 2010 (Bystricky et al. 2010) and 2012 (Roose et al. 2012). An increasing research interest has been observed since 2015 (Budzianowski and Budzianowska 2015; Patrizio et al. 2015). The geographical focus is on European countries (*n* = 16). There is a clustering for Italy (*n* = 5, e.g., (Cavana and Leone 2019; Patrizio and Chinese 2016)), Germany (*n* = 4, e.g., (Billig et al. 2019; Theuerl et al. 2019)) and Denmark (*n* = 2 (Fenton and Kanda 2017; Korberg et al. 2020)). Only three papers considered a region outside Europe: the USA (Murray et al. 2017), India (Bhatia et al. 2020) and Thailand (Wattanasilp et al. 2021). The research interest in Italy can be justified by the fact that the focus of biogas use has been set on biogas upgrading for use in the transport sector and a support program has been introduced since the Italian National Energy Strategy of 2017 (Murano et al. 2021).

There are different focuses on the upgrading of biogas in the literature, like the comparison of different biogas utilization pathways to the upgrading to biomethane (Horschig et al. 2019; Kalinichenko and Havrysh 2019; Wu et al. 2016), the potential role of biogas in the mobility sector of the future (Korberg et al. 2020) or the optimal process route, ranging from the upgrading technology to the transportation and utilization method, for biogas (Mohtar et al. 2021). The environmental impact can also be relevant to the profitability of the business model, CO_2_ prices can lead to higher costs for fossil fuels, so that biomethane becomes more profitable in comparison due to high GHG savings (Patrizio et al. 2017). National subsidies that contribute to the profitability of biomethane business models are also explored in the literature (Budzianowski and Budzianowska 2015; Patrizio and Chinese 2016).

The literature search produced comparatively few hits on the business model of H_2_ production. The five papers found are comparatively current from the years 2021 (Cvetković et al. 2021; Karaeva 2021; Wünning 2021) and 2020 (Antonini et al. 2020), only one publication dates back to 2013 (Wulf and Kaltschmitt 2013). No geographical focus of the research could be identified.

### Biogas business models

In the energy industry, especially in oil and gas production, a distinction is made between upstream and downstream business models. The upstream business models involve the identification, extraction or production of raw materials, while the downstream business models follow on from production and primarily involve sales to consumers (Bern 2012). This analysis will be limited to the downstream business models. The profitability is considered for agricultural biogas plants, biogas production by wastewater or organic municipal waste is not included in detail.

Possible business models are evaluated starting from the structure given by the three utilization pathways presented in Biogas utilization pathways. Several business models within each pathway were identified in the literature.

### Pathway 1: CHP generation

The literature review revealed that CHP use can be divided into two basic modes of operation, constant and flexible generation. Following this categorization, the results of the literature review are presented below. Flexible generation can be further categorized according to the goal of the flexibilization. The focus is usually on power-led operation (Bär et al. 2020; Lauven et al. 2019; Szarka et al. 2013), but heat-led operation is also being investigated (Ertem and Acheampong 2018; Güsewell et al. 2021).

### Business model 1.1: constant feed-in with heat concept

It is possible to generate a constant amount of energy in the CHP unit, the so-called baseload operation. Currently, about 75% of biogas plants in the EU use the biogas for CHP production (Calderón et al. 2021). The advantage of constant CHP generation over flexibilization is the low investment costs and the significantly lower effort required to operate the plant. However, the low costs do not usually lead to profitability, which would only be possible through subsidies (Lantz 2012). In comparison, the revenues in a flexible operation are always higher than in a baseload operation (Grim et al. 2015). As the European regulations show, the aim is not baseload operation as this is less beneficial to the energy system. Financial support is given to flexible power generation, with the aim of making the power from biogas usable for the system (Stürmer et al. 2021b). The long-term viability of this business model is, therefore, questionable.

### Business model 1.2: demand-oriented flexibilization

The second way to operate a CHP unit is to generate energy according to a demand or a price signal. Compared to other renewables that generate and feed in power in a volatile manner, biogas offers the possibility of being stored and thus generating energy flexibly and in accordance to demand (Szarka et al. 2013). In the case of flexibilization of biogas plants, the literature usually reports on flexibilization with the aim of its usage in the power market. By contrast, there is the possibility of a heat demand-oriented flexibilization of the biogas plant.

Thus, the goal is the balancing of volatile feed-in from other renewable energy sources such as wind turbines and PV plants (Güsewell et al. 2021), or the balancing of the residual load and the provision of system services (Szarka et al. 2013). In the energy system of the future, flexible power from biogas can reduce overall costs (Fleischer 2018) and can help operators generate additional revenue by selling power during periods of higher prices, potentially helping to make the plant profitable (Lauven et al. 2019). The revenues can be generated on the markets for ancillary services, e.g., as secondary or tertiary control reserve (Saracevic et al. 2018), or on the short-term markets, such as the day-ahead or spot market (Hochloff and Braun 2014).

The profitability of the business model is influenced primarily by the amount of the available overcapacities in comparison to the nominal load, schedule design and the amount of external heat use (Daniel-Gromke et al. 2019). For example, revenues can be increased by installing a thermal storage system (Wille-Haussmann et al. 2010). Furthermore, the amount of additional revenue is influenced by the size of the biogas storage capacity (Lauven et al. 2019). The business model of selling power is usually not profitable without subsidies due to declining income from power sales and the high investment costs in storage and CHP (Hochloff and Braun 2014; Lauven et al. 2019; Lee 2017). A positive net present value can be achieved with flexible operation of the existing CHP unit without an investment in new units under certain conditions (Grim et al. 2015).

The profitability of a CHP unit increases with high shares of heat utilization, e.g., by nearby industry or households (Goulding and Power 2013), so the idea of this utilization pathway is to raise the rate of heat utilization (of the externally available heat) to 100%, if possible (Güsewell et al. 2021). In the case of constant energy production by the CHP unit, a high utilization rate can only be achieved with a significantly higher heat demand than covered by the biogas plant (Güsewell et al. 2021). In the case of heat demand-oriented flexibilization, the biogas plant is designed and used in such a way that local heat sinks can be covered. Possible heat sinks can, for example, be owned by the biogas plant or neighboring agricultural companies, residential or public buildings, or agricultural drying processes (Herbes et al. 2018). The power that is generated simultaneously in the CHP unit is fed into the public power grid and remunerated according to the current tariffs (Daniel-Gromke et al. 2019). The actions to adapt an existing biogas plant to this utilization pathway are, firstly, the identification of potential heat sinks in the immediate proximity of the biogas plant, and, secondly, the adjustment of the generation profile to heat consumers. This can be realized by seasonal feeding, for example, adapted by the feedstock amounts used or substrates with different energy density. An alternative method is to use a biogas storage to compensate the fluctuations in the demand curve (Güsewell et al. 2021). The disadvantage is that many biogas plants are located in areas where heat utilization concepts are difficult to implement because potential consumers are too far away (Ertem and Acheampong 2018).

Short-term flexibilization increases profitability by exploiting higher power prices, while seasonal flexibilization additionally optimizes profitability by increasing heat utilization. However, both options currently do not lead to profitability in Germany under the current market conditions (Güsewell et al. 2021).

In addition to optimizing generation to meet an external demand, maximizing on-site energy use can also be a goal of flexibilization. Accordingly, it is necessary to adapt the generation profile of the biogas plant to the on-site load profile (Güsewell et al. 2020). The heat generated is currently already used for self-consumption or direct delivery. The idea of this utilization pathway is to consume the energy generated, i.e., power and heat, as completely as possible on-site or in the immediate vicinity. Possible sinks are the farm, residential houses or industrial plants. The aim here is to reduce energy purchase costs so that it is economically more advantageous to consume the energy produced by the biogas plant on-site instead of purchasing power from the grid or thermal energy from other external sources (Güsewell et al. 2020). The literature describes that favorable location factors are needed for this model to be profitable. These include low gas production costs and consumers close to the site with a permanently high demand for power. In addition, the possibility of the flexible control of consumer loads and a coupling with another depreciated renewable energy plant contribute to the advantageousness (Technische Hochschule Ingolstadt 2020).

The CO_2_ price will have a further influence on the profitability of CHP use in the future. Compared to CNG upgrading, CHP use leads to higher GHG savings, which, under favorable conditions, can lead to profitability even with a comparatively low CO_2_ price (Patrizio et al. 2015).

### Pathway 2: direct usage of biogas

The direct use of biogas can be divided into two categories: the use of biogas in households, for example, for cooking or heating, or in tractors or agricultural machinery. The use of biogas in households is mainly conducted in Asia or Africa, these family plants mainly pursue the aim to supply households with the needed energy and to replace firewood and dung as energy source (Kemausuor et al. 2018). Thus, these biogas plants are especially intended to provide an affordable and reliable source of energy for households (Hamid and Blanchard 2018). The use of waste can also be a major goal of a biogas plant (Kemausuor et al. 2018).

The biogas utilization in the mobility sector is not common practice without upgrading (Owczuk et al. 2019). There is the possibility of utilization in a dual-fuel engine (Jaber et al. 2009; Matuszewska et al. 2016; Owczuk et al. 2019), however, the use of biogas in engines is very limited, making this utilization pathway not sustainable as a business model.

### Pathway 3: upgrading of biogas to a gas of higher value

**Fig. 6:**
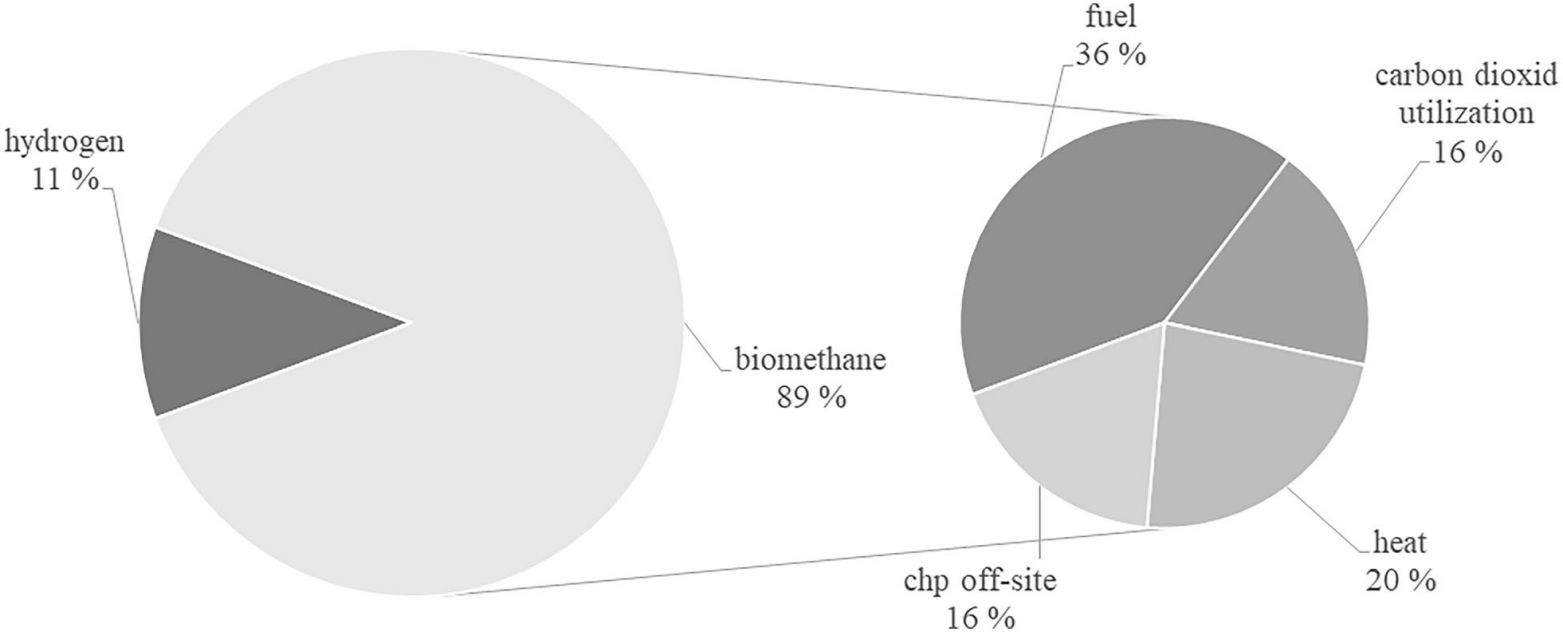
Share of the utilization pathways considered after biogas upgrading, *n* = 44

Biogas upgrading usage pathways can be divided into biomethane usage for CHP off-site usage, the heat market, mobility sector and on-site usage. Furthermore, there are the possibilities of CO_2_ usage and H_2_ generation. The central research interest has been the upgrading of biogas to biomethane, as shown in Fig. 6. Within biomethane utilization, its use as fuel is currently a focus of research, while the other utilization pathways are under development. The use of captured CO_2_ is a research interest that has emerged mainly in recent years.

The investment required for upgrading biogas into biomethane is higher than that for on-site power generation due to the plant technology required for upgrading (Deutsche Energie-Agentur GmbH 2019a). Upgrading biogas into biomethane is usually only an interesting business model when subsidies are involved since the costs for producing biomethane exceed the market price for natural gas (Budzianowski and Budzianowska 2015). However, rising natural gas prices may contribute to the better profitability of biomethane business models in the future (Banja et al. 2019). There are already various studies that evaluate the upgrading of biogas to biomethane as the best utilization path and assume that profitability can be achieved (Lee 2017).

### Business model 3.1: biomethane feed-in for CHP off-site utilization

Basically, power and heat from off-site biomethane CHP units can serve the same markets as on-site CHP units, for example for demand-oriented use or balancing the fluctuating generation of wind and solar power (Budzianowski and Budzianowska 2015). The advantage of this option is that the utilization is independent of the location where the biogas is produced, which can lead to higher efficiency due to a higher heat utilization rate, as well as the possibility to use the natural gas grid as biomethane storage (Budzianowski and Budzianowska 2015; Horschig et al. 2016; Szarka et al. 2013). In Germany, off-site CHP use is the most favored utilization path for biomethane with over 85%. The advantage here is the subsidy from the EEG (Renewable Energy Law) for the power generated by the CHP, which can lead to profitability and was so far the best stimulation for biomethane expansion (Daniel-Gromke et al. 2017). The range of the CHP units in which the biomethane can subsequently be used extends from 1 kW_el_ to 10 MW_el_. Thus, the use of biomethane is not only possible in an industrial context; mini CHP units in households are also available on the market (Fachagentur Nachwachsende Rohstoffe e. V. 2012). Since the price of biomethane is comparatively much higher than for natural gas and the infrastructure for its use causes the same costs, the substitution of natural gas by biomethane, e.g., in an industrial context, is not advantageous. Thus, profitability in comparison can only be achieved by the customer's decision to choose the more environmentally friendly option. In the future, pricing of CO_2_ emissions or alternatively the sale of green gas certificates can contribute to profitability (Patrizio et al. 2015).

### Business model 3.2: biomethane feed-in for the heat market

In addition to the use of heat from CHP units with a distribution via local heating networks, where the advantageousness depends on factors such as the proximity of the biogas plant to heat consumers and a suitable heat consumption profile, there is the possibility of providing heat via the use of biomethane (Banja et al. 2019). In this approach, biogas is upgraded to biomethane in upgrading plants and fed into the natural gas grid. Natural gas plays a central role in the heat supply particularly in countries with well-developed public natural gas grids, especially for supplying households and industry. This existing infrastructure can, thus, be used for the distribution of biomethane, since the latter can be substituted directly for the natural gas that is used throughout the country today (Cavana and Leone 2019). Since biomethane is a natural gas substitute, it can be used in conventional natural gas burners. Heating systems or gas stoves, therefore, do not have to be replaced (Banja et al. 2019; Fachagentur Nachwachsende Rohstoffe e. V. 2012).

In addition to its use in households, the use of biomethane is also interesting in industries that rely on the use of methane-based energy sources for heat generation, such as the iron and steel industry. Biomethane can reduce greenhouse gas emissions in these industries. However, according to current forecasts, an economic substitute is hardly possible even with rising CO_2_ prices (Ahlström et al. 2020). The profitability of biomethane production depends on plant size, substrate input (Cucchiella and D’Adamo 2016) and grid connection costs (Pasini et al. 2019), whereby the profitability of different plant constellations could definitely be proven (Cucchiella and D’Adamo 2016). The success of biomethane in the heating sector depends primarily on the prices of substitute goods and suffers, above all, from low natural gas prices. However, the trend of rising natural gas prices since 2016 could contribute to better profitability in the future (Banja et al. 2019).

Biomethane is currently provided at a higher price than natural gas even under the best circumstances (Paturska et al. 2015). In the private sector, therefore, the end consumers' willingness to pay is particularly decisive for the market success of biomethane. For this reason, utilities are currently focusing on gas tariffs with an admixture of biomethane (Dunkelberg 2015). Since there is no subsidy for the provision of biomethane in the heat market, e.g., comparable to feed-in tariffs in the power market (Herbes et al. 2021), a market ramp-up in this area is hardly to be expected (Adler et al. 2014).

CO_2_ prices will have a relevant impact on the success of biomethane in the future, especially in direct competition with natural gas. Compared to the direct use of biomethane in the mobility sector, the feed-in into the natural gas grid has the disadvantage that the GHG reduction is reduced by adding propane and thus can be monetized comparatively worse (Patrizio et al. 2015).

The issue of profitability is addressed in various studies, which conclude that the use of renewable gases by 2050 will be cheaper than full electrification of the heat sector (Cavana and Leone 2019). Other studies, including those which studied the use of biogas in gas boilers in Denmark and the EU, said that heat supply through district heating and individual electric heat pumps are better solutions compared to the use of biomethane. The reason is the more economical cost of the energy system and the biomass use being more economical in other sectors (Korberg et al. 2020).

### Business model 3.3: biomethane feed-in for the mobility sector

Another utilization pathway for biomethane is its use in the mobility sector. Here, it is possible to feed the upgraded biomethane into the existing natural gas grid and then make it available virtually at natural gas filling stations. This utilization pathway is already common today; mixed products of biomethane and natural gas are usually sold at gas stations (Fachagentur Nachwachsende Rohstoffe e. V. 2012). Compared to gasoline, the use of biomethane can save GHG emissions, reducing them by 60% when produced from corn, 70% when produced from waste and about 80% when produced from manure (Banja et al. 2019).

The future development of this sales channel depends on the development of CNG vehicles (Fachagentur Nachwachsende Rohstoffe e. V. 2012). However, biomethane can be used not only in passenger cars; there are already other vehicles that run on natural gas. These include, for example, light commercial vehicles, trucks, buses (Natural & Bio Gas Vehicle Association 2019) and ships (Backman and Rogulska 2016). The number of natural gas vehicles in Europe has been steadily increasing in recent years, reaching 1.46 million in 2020, of which 1.25 million are passenger cars. The market share of natural gas cars is the largest in Italy with 2.49% of the total stock of cars. Italy has the largest absolute number with 981,000 CNG-powered cars. Germany has the second largest number of CNG-powered cars in absolute terms (83,000), but they account for only 0.17% of the total car population (European Alternative Fuels Observatory 2020). Sweden has by far the largest share of CNG-powered buses with 17.6%, while the Czech Republic (6.7%) and the Netherlands (6.0%) also use CNG-powered buses (European Automobile Manufacturers Association 2021). Due to the partial lack of infrastructure in the area of natural gas filling stations and the associated investment costs, as well as the remaining GHG emissions, it is partly assumed that the future of mobility will instead be shaped by electromobility (Banja et al. 2019).

It has been shown that various influencing factors determine the profitability of biomethane use in the transport sector. These include primarily subsidies, but also plant size and substrates. Larger plants are generally more likely to be profitable, as is the use of residual and waste materials (Cucchiella et al. 2018; D'Adamo et al. 2019). Lower taxes for biomethane also offer advantages for profitability (Browne et al. 2011). Several studies have shown that biomethane can already be competitive with other fossil fuels or liquid biofuels (Goulding and Power 2013). Compared to liquefaction, injection into the natural gas grid is generally cheaper if connection costs are low (Pasini et al. 2019).

There is also the possibility of liquefying the biomethane at the points of use to produce bio-LNG (Liquified Natural Gas) (Hönig et al. 2019). Bio-LNG can also be used in the transport sector and is particularly interesting for ships or heavy-duty transport due to its higher energy density. Again, a large biogas plant and the use of residual and waste materials as substrates tends to be more profitable (Deutsche Energie-Agentur GmbH 2019b).

### Business model 3.4: decentral biomethane filling stations

As an alternative to feeding the biomethane into the natural gas grid, there is the possibility of setting up a gas filling station near the biogas or upgrading plant. The aim here is also to use the biomethane as a fuel in the mobility sector. This way has been less established so far, but there are already examples in Germany (Fachagentur Nachwachsende Rohstoffe e. V. 2012). Customers for the biomethane fuel in the proximity who have their own machines and vehicles, but also logistics companies or bus fleet operators are relevant for the success of this sales channel. In addition, there should be sufficient space for the construction of the upgrading plant and the biomethane filling station, and good accessibility for the customers should be ensured. If there are bottlenecks in the supply of biomethane by the biogas plant, a connection to the natural gas grid should be established (Grösch et al. 2020). This can also be used to feed in biomethane during periods of lower sales. Alternatively, it is necessary to ensure an alternative use or storage (Hornbachner et al. 2009).

### Business model 3.5: on-site usage of biomethane

In addition to the possibility of selling the biomethane produced, it can also be used on-site. The possibilities for use are similar to the on-site use of biogas (Pathway 2) and are primarily intended to contribute to reducing energy procurement costs and increasing the degree of energy self-sufficiency. Thus, biomethane can be used as a fuel in agriculture, replacing diesel and biodiesel. This has a high potential to reduce GHG emissions, contribute to a higher share of renewable energy in the agricultural sector and, thus, to a more sustainable agriculture (Bisaglia et al. 2018). Initially, there were tractors that were converted to use biomethane, and dual systems for the joint use of diesel and biomethane were also used (Bisaglia et al. 2018). In the meantime, a first series tractor that runs 100% on biomethane has reached market maturity (New Holland Agriculture UK 2021). However, the business model is not widespread, so there is no reliable information on profitability.

### Business model 3.6: CO_2_ utilization

Carbon dioxide is captured during the upgrading of biogas to biomethane. While the captured CO_2_ is currently usually released into the atmosphere and not used further, it is also possible to use the captured CO_2_ as well. This offers the opportunity in the field of carbon capture and utilization to make a further contribution to climate protection and replace fossil CO_2_ generation (Billig et al. 2019). There are different ways to use and monetize CO_2_. Large amounts of dry ice are used, especially in the food industry, dry ice service companies and the chemical and pharmaceutical industries (Horschig et al. 2019). There are already initial studies showing that it is possible to produce CO_2_ for food market utilization. Since the food market offers the most restrictive quality requirements, it would be therefore also conceivable to produce CO_2_ for other markets. The CO_2_ can also be used, for example, in Fischer–Tropsch synthesis for the production of cosmetic products or for the production of high-value chemicals and waxes (Horschig et al. 2019). It is also possible to use CO_2_ to produce synthetic fuels. Power-to-fuel, for example, can use carbon to produce renewable fuels together with H_2_ via methanol synthesis (Eggemann et al. 2020). By selling the CO_2_, the joint use pathway with biomethane can become profitable (Esposito et al. 2019). Alternatively, the capture of CO_2_ can also lead to negative CO_2_ emissions in biogas production. The BECCS process is considered profitable (Li et al. 2017). If regulations in the area of the carbon emission trading systems are strengthened, additional revenue can be generated through the negative CO_2_ balance (Carranza-Abaid et al. 2021; Lisbona et al. 2021).

### Business model 3.7: hydrogen production

Green H_2_ is central in the discussion about energy transition. Comparing the possibilities of producing H_2_ from biogas, steam reforming leads to twice the H_2_ yield compared to power generation with subsequent electrolysis (Wünning 2021). The H_2_ is currently transported via trucks as there is no existing H_2_ grid. An alternative to this could be the decentralized production of biomethane with feed-in to the natural gas grid and subsequent decentralized upgrading to H_2_. On the other hand, decentralized production can also be an advantage for the nationwide production and distribution of H_2_ for use in the transport sector (Wünning 2021). In the long term, it is being discussed whether the natural gas infrastructure can be used to distribute H_2_ leading towards the elimination of the use of fossil fuels (Dodds and Demoullin 2013).

Due to the existing technology of the steam reforming, which already produces hydrogen by using natural gas, it is a market-ready and profitable technology. Compared to using natural gas, biomethane is more expensive but can also result in negative GHG emissions, which in turn can contribute to profitability (Braga et al. 2013). The payback period is less than 10 years (Braga et al. 2013; Montenegro Camacho et al. 2017) and the net present value is positive (Yao et al. 2017), making it possible to operate profitably over its lifetime. With production costs below 5 €/kg, biogenic hydrogen is considered competitive (Marcoberardino et al. 2018; Montenegro Camacho et al. 2017).

The sales markets for hydrogen are diverse and will continue to grow in the future. In industry, hydrogen is used, e.g., in refineries, in chemical production or in the future also in the steel and iron industry, as these will renounce the use of natural gas and coal in order to decarbonize. Furthermore, hydrogen is suitable for generating high-temperature heat (Noussan et al. 2021). In the transport sector, there are already passenger cars that run on hydrogen (Alves et al. 2013; Antonini et al. 2020). In the future, however, the need is seen more in applications that are difficult to electrify, such as trucks, buses, ships and aircraft. Hydrogen is also expected to play a relevant role in the energy system for flexible power generation in the future (Noussan et al. 2021).

## Discussion

There has been research interest in utilization pathways and business models in the field of biogas plants since 2004. However, this interest has increased steadily only since 2009. Considering that biogas plants were already being built in European countries in the 1980s (Demuynck and Nyns 1984), it is initially surprising that the research interest has only increased strongly in the last decade. However, during the period of this development, new requirements of the energy system have also emerged.

The systematic literature research has shown three basic ways of utilization: the utilization of biogas in a CHP plant, the direct utilization of biogas and the upgrading to a gas of a higher quality. These three utilization pathways contribute in different sectors. Flexible generation of biogas (Business model 1.2: Demand-oriented flexibilization), for example, could provide a system service in the power market by generating power from biogas during hours of low power generation from wind and photovoltaics. Many papers have already analyzed this business model extensively. However, compared to other renewables, biogas has high power generation costs and different studies conclude that it is unlikely to be competitive in the long term. On the other hand, it has also been shown that flexible power generation can be profitable under beneficial conditions, such as a high thermal and biogas storage. Another important influence are rising power prices due to rising costs of fossil fuels and rising CO_2_ prices due to high GHG savings in CHP usage. Furthermore, the development of technologies to provide system services in the power market will have a relevant influence on the biogas usage in the future. A distinction has to be made between technologies for the short-term and long-term provision of flexible energy. It can be assumed that power storage and flexible generation from H_2_ and biogas will complement each other.

Another option that has been considered in the literature is the direct use of biogas (Pathway 2: Direct usage of biogas). The option of using biogas in tractors and machinery does not seem very promising, as the lower energy density of biogas does not lend itself well to mobile applications. The use in households for heating, cooking and lighting is limited to low-income countries in Asia and Africa, where the energy supply is not sufficiently developed, especially in rural areas. In Europe, however, the focus is on CHP generation. Therefore, it is not expected that direct use of biogas will be a relevant business model in the future.

The last option is the upgrading of biogas (Pathway 3: Upgrading of biogas to a gas of higher value). The use of biomethane offers the advantage of versatility and distribution via an existing infrastructure. Many papers consider possibilities and potentials of the use of biomethane and compare it to alternative use pathways for biogas. A special focus is on the use in the mobility sector. The use of biomethane is of particular interest for vehicles that cannot be powered by power, such as ships or aircraft. The profitability of the use of biomethane in the various pathways currently depends on subsidies or the consumer decision for a green product. The future development in the area of GHG neutrality is relevant here. In a GHG-neutral energy system, biomethane can be relevant to cover methane-based needs in an industrial context. However, a carbon–neutral energy system of the future cannot work with natural gas, as it generates GHG emissions when used. Therefore, the long-term perspective of using the natural gas grid for biomethane is uncertain. In the long term, it could be advantageous for biogas plants to cover regional methane demands with biomethane, for example in the mobility sector in the form of filling stations or industrial demands.

Another advantage of upgrading biogas to biomethane is that the captured CO_2_ can be made usable, thus, generate additional revenue. This CO_2_ can be used as a substitute for fossil-generated CO_2_. A contribution in the area of carbon capture and storage would also be conceivable here, so, CO_2_ could be specifically absorbed in cultivated plants, which would then be used in biogas plants. The CO_2_ separated in the upgrading process could then be stored and, thus, removed from the environment. Consequently, biogas plants could contribute to energy production and the reduction of CO_2_ in the atmosphere. The separation and utilization of CO_2_ can contribute to the profitability of biomethane use in the future. This utilization pathway is influenced by the acceptance of use of biogenic CO_2_, e.g., in the food industry, and the costs and revenues of CO_2_ storage.

The topic of generating H_2_ from biogas (Business model 3.7: hydrogen production) is still of little consideration in the literature from the point of view of business models. However, since the technology for upgrading is already available, this utilization pathway would be feasible in the short term. Biogas could find a long-term sales market in a hydrogen based energy system. First studies show that biogenic H_2_ can be profitable and competitive. Here, however, the competitiveness has to be investigated in detail.

A major unknown component for the future development of business models for biogas plants is national legislation. This can lead to a certain utilization pathway becoming financially more attractive. In the EU, for example, the Renewable Energy Directive 2018/2001 (RED II) was issued in 2018, which aims to achieve the climate protection targets—a GHG reduction in transport of 40 to 42% by 2030. Therefore, there should be a share of at least 14% renewable energy in fuel consumption by 2030, as well as a sub-quota of 3.5% for advanced biofuels from residues such as straw and manure (European Parliament and Council of the European Union 2018). Biomethane from residues can play a particularly important role in meeting the sub-quota. In addition, financial support for biogas plants in the form of feed-in tariffs for power or biomethane or investment subsidies is also conceivable. This financial support can be differentiated so that, for example, special substrates or technical processes are supported. Tax benefits are also conceivable, giving a competitive advantage to renewables over fossil alternatives, as it is already common practice in Sweden in the mobility and heating sectors. These tax benefits may be tied to minimum GHG reduction standards, for example.

A major influence in the future will be the CO_2_ price, which will contribute to the profitability of various potential biogas utilization paths in the long term. On the one hand, the CO_2_ price can ensure that fossil alternatives become financially less attractive compared to biogas and, on the other hand, additional revenues can be achieved in the production and sale of biogas through the sale of certificates. Accordingly, the further development of legislation is a major factor influencing the development of the biogas industry.

## Conclusion

The use of biogas and associated business models are discussed worldwide, but with a clear dominance of the European perspective. Outside of Europe, the focus is on basic potential analyses of the use of biogas, and in Asia and Africa, also on the use of biogas in households. This business model is not discussed for Europe. The majority of the papers was published within the last decade. During this time, the number of publications has increased steadily, but not explosively.

Furthermore, a development in the focus of the discussion of business models has emerged. The consideration of the direct use of biogas has been relatively constant over the years at a comparatively low level. In Europe, CHP generation is currently the dominant utilization pathway, with an increasing focus on flexibilization since 2015. The dominant aim of flexibilization was to satisfy a power demand. Research on business models for biogas utilization has evolved since 2019, and the focus is now particularly on the upgrading of biogas. Regarding upgrading, many new utilization pathways and business models for biogas have arisen in Europe, after a long period of mainly on-site CHP use. Biogas is becoming more valuable and even more diverse in its possible uses through upgrading. Upgrading biogas to H_2_ still represents a small part of the relevant research literature. It is not surprising that research into the field of business models for upgrading biogas to H_2_ has been increasing in recent years especially with the increasing demand for H_2_ expected in the future.

The systematic literature research has shown that no business model proves profitable under all circumstances, so attention must generally be paid to the individual case of the biogas plant. Direct use is only a partially interesting business model in regions without a well-developed gas distribution infrastructure. The possibilities of flexible CHP use and upgrading appear promising in the literature. Future use of CHP is only viable with high flexibility. If fossil options for flexible generation are abandoned in the future, the synergies of different flexibility options can be used to provide system services. Thus, biogas plants can play a crucial role, especially in the power sector of the future. On the other hand, upgrading biogas to biomethane offers the advantage of multiple uses. Especially in applications where there are no other green alternatives, biomethane could play a decisive role in the future. For example, biomethane can meet methane-based needs in industry or serve as a fuel for heavy-duty transportation. In the future, however, biomethane upgrading should always be accompanied by CO_2_ capture and use or storage, with the goal of increasing profitability and environmental benefits. Upgrading of biogas to hydrogen has not been investigated much yet, but could play a significant role in the future. Further investigations are needed to determine whether upgrading to hydrogen has any advantages. Factors influencing the profitability of a given business model thus include the size of the biogas plant, since upgrading biogas becomes more cost-effective especially as volumes increase, and the availability of local customers for heat or generated biomethane.

The development of the CO_2_ price will be relevant for biogas plants in the future. On the one hand, this has an influence on the amount of revenues by trading certificates for the reduction of GHG emissions and, on the other hand, on the competition with fossil fuels, which become comparatively more expensive due to a higher CO_2_ price, resulting in a competitive advantage for biogas. However, as various studies have shown, political support instruments are particularly relevant for a development of a country's biogas plant stock. Thus, the promotion of a certain business model leads to an increasing implementation of this utilization pathway. In the interest of the national GHG reduction strategies, it would be useful to set clear signals for the future development of existing plants, so that plant operators can develop a clear long-term strategy for the use of their plants.

The utilization pathways for biogas have become more diverse and each of these pathways has advantages. However, there is competition between the individual pathways since biogas as an energy resource is finite. The future political framework is a particularly relevant influencing factor on the development of the biogas industry. It has already led to the biogas industry developing differently in different countries as a result of various support programs. It needs a consistent political framework for a target-oriented, further development of the industry in the next few decades. The system utility of the use pathways can be a major argument for use in a particular energy sector. The environmental impact of biogas use will also have a greater influence on utilization decisions in the future; the goal of integrating it into a circular economy is already being explored.

## Data Availability

The datasets used and/or analyzed during the current study are available from the corresponding author on reasonable request.
